# Phthalate and Metal Concentrations in Drinking Water in Lagos, Nigeria

**DOI:** 10.5696/2156-9614-8.18.30

**Published:** 2018-06-11

**Authors:** Ebenezer Olasunkanmi Dada, Rose Kasuwa Ikeh

**Affiliations:** Department of Cell Biology and Genetics, Environmental Biology Unit, Faculty of Science, University of Lagos, Akoka, Yaba, Lagos, Nigeria.

**Keywords:** Contamination, dibutyl phthalate, diethyl phthalate, dimethyl phthalate, regulatory bodies

## Abstract

**Introduction.:**

There have been no studies, monitoring programs, or data about phthalate levels made available to the public on the safety of residential drinking water in Lagos, Nigeria.

**Objectives.:**

The present study aimed to assess the concentrations of dimethyl phthalate (DMP), diethyl phthalate (DEP), dibutyl phthalate (DBP), and zinc (Zn), lead (Pb), chromium (Cr), and cadmium (Cd) in drinking water drawn from taps in three residential areas of the state.

**Methods.:**

High performance liquid chromatography and atomic absorption spectroscopy were used to determine the concentrations of phthalates and metals, respectively.

**Results.:**

All of the water samples collected throughout the sampling period contained DMP, while DEP and DBP were present in only some of the samples. The highest mean DMP, DEP, and DBP concentrations of 1.15±0.28 mg/l, 0.09±0.16 mg/l, and 0.28±0.33 mg/l, respectively, were found in water samples collected from Lagos Street (Ebute-metta East). In addition, the trace/toxic metal concentrations in the water samples were found to be low for Cr, but high for Cd, Pb, and Zn, especially when compared with World Health Organization (WHO) limit values for drinking water. Lead recorded the highest mean concentration of 0.087±0.021 mg/l in the water samples obtained from Apapa Road (Ebute-Metta West). In the same vein, the highest significant (P < 0.01) mean Cr concentration of 0.047±0.012 mg/l was found in the water samples obtained from Apapa Road (Ebute-Metta West).

**Conclusions.:**

In view of the high concentrations of phthalates and metals in the water sampled in this study, and the potential adverse health effects of these contaminants, especially on children and women of child-bearing age, the Lagos State Government of Nigeria and the state water corporation are called upon to immediately institute a monitoring program to identify the sources of contaminants and take appropriate intervention measures.

**Competing Interests.:**

The authors declare no competing financial interests.

## Introduction

Because it is a universal solvent, water is potentially exposed to many contaminants in the environment. In most waterworks, many contaminants, including chlorides, nitrates, total coliform, and metals are regulated and tested for in drinking water to ensure the safety of drinking water. However, there are some contaminants such as pharmaceuticals and phthalates, whose adverse effects on health have been established, but are not readily captured in the routine drinking water standards released by regulatory bodies.^1^,^2^,^3^ The lack of drinking water standards should not preclude, nor be used as a reason for not taking action to eliminate or minimize exposure to phthalates or any other contaminant found to pose a risk to public health.

The present study aimed to assess the concentrations of some phthalates, namely dimethyl phthalate (DMP), diethyl phthalate (DEP), dibutyl phthalate (DBP) and trace/toxic metals zinc (Zn), lead (Pb), chromium (Cr), and cadmium (Cd) in water drawn from taps in three residential areas of Lagos, Nigeria.

### Phthalates

Phthalates are a group of chemicals added to products to give them flexibility, durability, and other desirable properties. They are among the most preferred plasticizers used in plastic products, especially polyvinyl chloride (PVC) plastics. Phthalates are also used in products such as children's toys, paints, textiles, food packaging, dental materials, perfumes, nail polish and other cosmetics.^4^,^5^,^3^ Because phthalates are not covalently bound to the polymer matrix of plastics, they are prone to migrating (leaching) out of their polymer matrix to the surrounding air, soil or water bodies, where they become contaminants.^6^,^7^,^8^ Water bodies are especially open to phthalate contamination because up to one percent of the phthalate component of plastic materials may be released into the aquatic environment.^9^ Excessive exposure to phthalates, especially through drinking water, may result in adverse health conditions including endocrine system disruption, cancer, developmental abnormalities, and polynueropathy.^4^,^10^,^11^ The United States Environmental Protection Agency (USEPA) has set threshold limit values of 0.55, 0.45, 5.0, and 5.0 mg/l for DEP, DBP, DMP, and diethylhexyl phthalate (DEHP), respectively.^12^ The USEPA has also listed some phthalates as priority pollutants, including DMP, DEP, di-n-butyl phthalate (DnBP), di-n-octyl phthalate (DnOP), butyl benzyl phthalate (BBP), and DEHP.^13^ There is a need for efforts to monitor these and other phthalates in the environment, especially in drinking water.

### Metals

Metal contamination in drinking water has been a subject of concern for sometime. While some metals like Zn, copper (Cu), cobalt (Co), manganese (Mn) are classified as essential trace elements, others like Pb and Cd have no known biological functions. The World Health Organization (WHO) and other water regulatory agencies have established permissible levels for metals and other contaminants based on human health risks. Sustained disregard for these limits could result in various adverse health effects. Irrespective of the type of metal, above certain concentrations, they are toxic to humans, as they accumulate in vital organs like the brain, kidney, and bone. Depending on the type of metal, the health effects of metal poisoning may include cancer, paralysis, gastrointestinal disorders, vomiting, kidney dysfunction, anemia, and hypertension. Cadmium, Cr, Pb, and Zn are contaminants commonly found in corroding galvanised pipes. Lead contamination poses a substantial public health risk, especially to the development of children.^14^,^15^,^16^,^17^

Depending on the health of an individual, up to 20% of ingested Cd may be absorbed through the gastrointestinal tract. Although the adult human body has a limited capacity to mitigate the toxic effects of Cd and some other metals through the sequestering actions of metallothioneins, when this detoxifying mechanism is overwhelmed, Cd accumulates over time, mainly in the kidney and liver, resulting in their malfunctioning.^16^

Lead is also a cumulative general poison capable of causing serious disruption of the human nervous system. Acute and long-term exposure to Pb can result in intoxication manifesting in dullness, restlessness, irritability, poor attention span, kidney damage, memory loss, and insomnia. Children are especially vulnerable to these adverse effects because their body's contaminants detoxifying systems are not yet fully developed. The WHO notes that consistent exposure of infants, children, fetuses, and pregnant women to even low levels of Pb can impair neurodevelopment, leading to low IQ, irritability, and poor attention span.^17^ Excessive exposure to Zn has been found to result in adverse health conditions such as pulmonary distress, fever, chill, and gastroenteritis.^15^

**Abbreviations**
*DBP*: Dibutyl phthalate
*DEHP*: Diethyl hexyl phthalate
*DEP*: Diethyl phthalate
*DMP*: Dimethyl phthalate
*HPLC*: High performance liquid chromatography
*USEPA*: United States Environmental Protection Agency
*WHO*: World Health Organization

## Methods

### Collection of water samples

**Figure 1:**
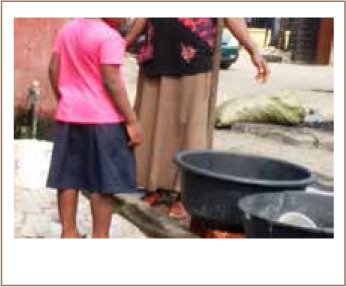
Water being drawn from one of the sampling taps

The water samples used for the present study were drawn from public water supply taps (*Figure 1*) in three residential areas of Lagos State, Nigeria: (1) Surulere, Latitude 6.2994°N; Longitude 3.2157°E, (2) Lagos Street (Ebute-metta East), Latitude 6.2925°N; Longitude 3.2289°E), and (3) Apapa Road (Ebute-mettaWest), Latitude 6.2889°N; Longitude 3.2244°E, all in the mainland area of the state. Water was collected from each of the taps once a week for three consecutive weeks. Each water sample was collected in a 1 liter polyethylene bottle. The polyethylene bottles are dedicated bottles that had earlier tested negative for phthalates when filled with blank samples. Bottles were washed with deionized water prior to sample collection. After sample collection, the bottles were immediately taken to the laboratory where they were refrigerated in the dark at 4°C to avoid contamination and degradation before analysis.^18^

The concentrations of phthalates in the water samples were quantified using high performance liquid chromatography (HPLC) (Shimadzu Nexera MX).

### Extraction

Extraction was carried out by measuring 20 ml of the water sample into a separation funnel and adding 20 ml of acetonitrate/methanol (3:1). The mixture was shaken vigorously for 30 minutes, while releasing the funnel cap intermittently to release pressure build-up. The aqueous end was run off while the hydrocarbon (non-polar) end was collected in a 25 ml standard flask and made up to the mark with acetonitrate/methanol solution. This was used for phthalate analysis.

### Analysis

A standard form of individual pure phthalate of known concentration was first injected into the HPLC, generating a chromatogram with a given peak area and peak profile. These were used to create a window in the HPLC in preparation for the test sample analysis. An aliquot of the extracted test sample was also injected into the HPLC to obtain a corresponding peak area and peak profile in a chromatogram. To obtain the sample concentration, the peak area of the sample was compared with that of the standard, relative to the standard concentration. The HPLC was set to accommodate the dilutions made during extraction. Equation 1, shown below, was used to calculate the concentration of the sample.


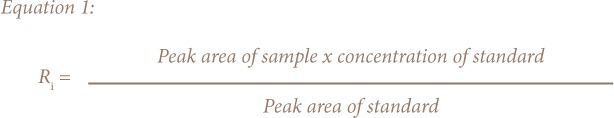


### Programming of HPLC

Column (stationary) phase: uBondapak C18; length = 100 mm; internal density = 4.6 mm; thickness = 7 μm; mobile phase: acetonitrate/water (3 :1); sample injection volume: 5 ml; flow rate: 0.08 ml/minute (W); 5 ml/minute (A); detector: UV e 254 nm; temperature: room temperature (29°C).

### Quantification of metals in water samples

To determine the concentrations of metals in the water samples, 20.0 ml of water sample was measure into a conical flask. Dilute nitric acid (HNO_3_) (HNO_3_: deionized water = 1 : 3) was added to the sample in a conical flask and heated in a Bunsen burner until all the reddish yellow fumes were expelled. The solution was brought down, allowed to cool, and transferred into a 10 ml standard flask. The standard flask was made up to mark with distilled water. Analysis of metal content was then carried out using an atomic absorption spectroscopy (AAS) (Perkin Elmer model 460).

A suitable standard curve was generated in the AAS using standard solutions of metals of interest. Standard concentrations of 6, 12, 18, 24, 30 mg/l were used. Adequate standard was generated for each metal passing through the origin. After a good curve was achieved, the digested sample solutions were aspirated into the AAS for the analysis. A unique cathode lamp for each metal was utilized for the analysis, operating at its peculiar maximum operational wavelength. A digital concentration read-out (meter) gave the direct concentration of the metals as contained in the test sample. The dilutions made during metal extraction were factored into the calculations of metals in the water samples.

### Statistical analyses

The data obtained for each phthalate and metal type were tested for homogeneity of variance and subsequently processed by one-way analysis of variance (ANOVA) to determine the differences in contaminant concentrations between sampling locations. The mean differences were compared for statistical significance using the least significant difference method (post hoc test). All statistical analyses were carried out using the Statistical Package for the Social Sciences software (SPSS) version 20.

## Results

### Phthalate concentrations in tap water

All the water samples collected for assessment contained different concentrations of phthalates (*Table 1*). The phthalates were DMP, DEP, and DBP. Dimethyl phthalate was consistently present in all of the water samples collected from the three locations throughout the sampling period, while DEP and DBP were present in some locations only. In addition, DMP occurred in high concentrations relative to the other phthalates, DEP and DBP, in all of the water samples. Water samples drawn from the tap in Lagos Street (Ebute-metta East) had the highest mean DMP, DEP, and DBP concentrations of 1.15±0.28 mg/l, 0.09±0.16 mg/l, and 0.28±0.33 mg/l, respectively. The water drawn from the tap in Surulere had the least mean DMP concentration of 0.81±0.21 mg/l. There were no significant differences (P > 0.05) in the concentrations of phthalates in the water samples taken from the three residential areas (*Table 2*).

**Table 1:** Phthalate Concentrations in Tap Water Across Study Locations

		**Phthalate concentration (mg/l)**			
**Phthalate type**	**Sampling location**	**Week 1**	**Week 2**	**Week 3**	**Mean+SD**
***DMP***	Surulere	0.64	0.75	1.05	0.81±0.21
	Lagos St.(Eb. East)	1.47	0.98	0.99	1.15±0.28
	Apapa Rd.(Eb. West)	1.19	1.04	0.91	1.05±0.14
***DEP***	Surulere	ND	0.12	ND	0.04±0.70
	Lagos St.(Eb. East)	0.27	ND	ND	0.09±0.16
	Apapa Rd.(Eb. West)	ND	ND	ND	ND
***DBP***	Surulere	ND	ND	0.18	0.06±0.10
	Lagos St.(Eb. East)	0.65	ND	0.21	0.28±0.33
	Apapa Rd.(Eb. West)	0.28	ND	ND	0.09±00.16

Abbreviations: Eb. East, EbuteMetta East; Eb.West, Ebute-Metta West; ND, not detected.

**Table 2:** ANOVA of Phthalate Concentrations in Water Samples Across Study Locations

**Source**	**Variation type**	**SS**	**df**	**MS**	**F**	**Sig**
***DMP***	Between group	0.176	2	0.088	1.840	0.238
	Within group	0.286	6	0.048	-	-
***DEP***	Between group	0.012	2	0.006	0.629	0.565
	Within group	0.058	6	0.010	-	-
***DBP***	Between group	0.090	2	0.045	0.917	0.449
	Within group	0.294	6	0.049	-	-

Abbreviations: SS, sum of squares; df, degree of freedom; MS, mean square; F, F-ratio; Sig, level of significance.

### Trace/toxic metals concentrations in water samples

The trace/toxic metal concentrations in the water samples were found to be low for Cr, but high for Cd and Pb, especially when compared with WHO limit values for drinking water (*Table 3*). The analysis of variance (*Table 4*) revealed that the differences in mean concentrations of Cr in the water samples from the three locations were significant (P < 0.05). Zinc recorded the highest mean concentration of 3.050±0.523 mg/l in the water samples drawn from the tap in Surulere, while Pb recorded the highest mean concentration of 0.087±0.021 mg/l in water samples obtained from Apapa Road (Ebute-Metta West). In the same vein, the highest significant (P < 0.01) mean Cr concentration of 0.047±0.012 mg/l was found in the water samples obtained from Apapa Road (Ebute-Metta West).

**Table 3:** Trace/Toxic Metal Concentrations in Water Samples Across Study Locations

		**Trace/Toxic metal concentrations (mg/l)**				
**Metal**	**Sampling location**	**Week 1**	**Week 2**	**Week 3**	**Mean+SD**	**WHO**
***Zn***	Surulere	3.288	3.411	2.450	3.050±0.523	Not given
	Lagos St.(Eb. East)	2.818	3.141	3.045	3.001±0.116	
	Apapa Rd.(Eb. West)	2.571	2.724	2.846	2.714±0.138	
***Pb***	Surulere	0.020	0.050	0.050	0.040±0.173	0.010
	Lagos St. (Eb. East)	0.080	0.003	0.040	0.041±0.039	
	Apapa Rd. (Eb. West)	0.110	0.080	0.070	0.087±0.021	
***Cr***	Surulere	0.001	0.002	0.001	0.001±0.001	0.050
	Lagos St. (Eb. East)	0.003	0.003	0.002	0.002±0.001	
	Apapa Rd. (Eb. West)	0.060	0.040	0.040	0.047±0.012^*^	
***Cd***	Surulere	ND	ND	ND	ND	0.003
	Lagos St. (Eb. East)	0.001	ND	0.001	0.001±0.001	
	Apapa Rd. (Eb. West)	0.005	0.003	0.001	0.003±0.002	

^*^Significantly higher (P < 0.01) than the mean concentrations of the same metal from other sampling locations.

Abbreviations: Eb. East, Ebute-Metta East; Eb. Wes, Ebute-Metta West.

**Table 4:** ANOVA for Trace/Toxic Metal Concentrations in Water Samples Across Study Locations

**Source**	**Variation type**	**SS**	**df**	**MS**	**F**	**Sig**
***Zn***	Between group	0.198	2	0.099	0.928	0.445
	Within group	0.640	6	0.107	-	-
***Pb***	Between group	0.004	2	0.002	2.886	0.132
	Within group	0.004	6	0.001	-	-
***Cr***	Between group	0.003	2	0.002	13.161	0.008
	Within group	0.001	6	< 0.001	-	-
***Cd***	Between group	< 0.001	2	< 0.001	5.154	0.50
	Within group	< 0.001	6	< 0.001	-	-

Abbreviations: SS, sum of squares; df, degree of freedom; MS, mean square; F, F-ratio; Sig, level of significance.

## Discussion

The presence of DMP, DEP, and DBP in the water samples assessed in the present study is an indication of phthalate contamination in the area's residential drinking water. The fact that there were no significant differences in the concentrations of phthalates in the water samples obtained from the three study areas suggests source contamination or local contamination occasioned by similar prevailing factors. The average concentrations of these phthalates in the water samples were below or within the range of the threshold limit values set by the USEPA (1991).^3^ However, assessing toxicity based on each phthalate separately may underestimate the potential risk of phthalates, as individuals are usually exposed to more than one type of phthalate at the same time and from multiple sources. The adverse effects not seen with individual phthalates may begin to manifest with exposure to multiple phthalates.^19^ Phthalate contamination in treated drinking water has also been reported in other countries such as the US, Japan, and China.^1^,^2^ Li *et al*. analysed six treated and untreated drinking water samples for presence of chemicals and found no significant differences in the concentrations of DBP between the treated and untreated water samples.^19^ The trend of phthalate contamination in drinking water calls for concern as excessive exposure to phthalates has been linked to several health conditions, including endocrine system disruption, cancer, developmental abnormalities, and polynueropathy.^4^,^10^,^11^ Water treatment plants and companies should adopt new approaches to water treatment to control and minimise the levels of phthalates and other contaminants in drinking water.

The most effective remediation and control strategy involves reducing phthalate usage in plastics and other products from which phthalates migrate into water bodies and other components of the environment.^6^,^7^,^8^,^9^ This can be achieved by replacing phthalate use in products with alternatives more friendly to the environment and human health, such as acetyl tri-n-butyl citrate, di(2-ethylhexyl) adipate, trioctyltrimelliate, dipropylene glycol dibenzoate, phosphate esters, sebacate and azelate esters, and others listed by the USEPA.^3^ Other potential substitutes for phthalates are low molecular weight polymeric ester plasticizers derived from polymeric multifunctional alcohols and adiphic, sebactic or glutaric acids, polymeric rubbers and plastics.^3^,^20^

In addition to source reduction strategies for controlling phthalate pollution, another potential remediation approach is enhanced or facilitated microbial decomposition. Microbial degradation has been described as one of the major routes of removal of phthalates from the soil, air, and water environments.^13^ Phthalates are naturally biodegradable. The general biodegradation and mineralization pathway for phthalates consists of primary biodegradation from phthalate diesters to phthalate monoesters, and then to phthalic acid. Phthalic acid is eventually biodegraded to either CO_2_, methane, or both.^9^,^21^,^22^ Some bacteria strains have been found to have the ability to facilitate this process, thereby helping to remove phthalates, especially DBP, DEP, and diethylhexyl phthalate (DEHP) at a faster rate from the environment. These bacteria include Gordonia spp., Agrobacterium spp., Variovorax spp., Bacillus sp. and Gordona sp.^13^,^23^,^24^ Water treatment plants and companies in Nigeria and elsewhere should consider adapting this natural bacterial remediation process into their routine water treatment processes. Suitable isolates of bacteria strains can be inoculated in water treatment reservoirs, individually or in combination, to bring about phthalates reduction in drinking water before the final chlorine treatment is carried out. This will go a long way towards reducing exposure to phthalates through drinking water.

In the meantime, if these results are confirmed and the source of phthalate contamination is traced to a water supply source, an immediate simple remediation strategy involves changing the water supply source for those residential areas. Meanwhile, since two flow rates were programmed for HPLC in the analysis of phthalates, this might affect the retention times and perhaps, by extension, the peak area/peak profile. Therefore, subsequent studies are needed to confirm the reproducibility of the current results.

The trace/toxic metal concentrations in the water samples were found to be low for Cr, but high for Cd and Pb, especially when compared with WHO limit values for drinking water. Lead was found in all of the water samples drawn from the three residential areas at levels that exceeded the WHO drinking water standards of 0.010 mg/l.^17^ Cadmium was consistently found in samples collected at Apapa Road (Ebute-Metta West), and in two of the three samples, the levels equalled or exceeded the WHO drinking water standards of 0.003 mg/l.^16^ To our knowledge, there have been no prior existing studies, monitoring programs, or data made available to the public about the levels of phthalates in drinking water from Lagos public taps. Effective contaminant control and regulation procedures to ensure the safety of drinking water should be implemented, just as in other countries where metals and other contaminants including chlorides, nitrates, total coliform are regulated and tested for in drinking water.^1^,^2^,^3^ The high concentrations of Cd, Pb, and Zn found in the water samples in the present study are greatly concerning. Although Cr occurred in low concentrations in the water samples, its synergistic actions with other metals and phthalates may be more serious than the health effects of each individual contaminant, even at those low concentrations.

The high concentrations of Cd, Zn, and Pb (and perhaps phthalates) found in the water samples in the present study cannot be dissociated from the effects of the deteriorating galvanized pipe network. This assumption stems from the fact that the supply pipes and taps from which the water samples were drawn either showed signs of deterioration or breakage. In one of the sampling locations, residents took turns drawing gushing water from the broken discharge pipe that had no tap and opened into the visibly dirty gutter. Moreover, in some parts of Lagos outside of the sampling areas, it is not uncommon to see broken pipes gushing water onto the roads, streets, and drainages. It is also not unusual to see a film of brownish red coloration forming on the surface, or fine particles settling on the bottom of tap water left in a bucket overnight. Furthers studies are needed to confirm the findings and assumptions of the present study. Moreover, additional residential areas supplied by different water treatment plants need to be sampled to sufficiently assess the quality and safety of water delivered to the people of Lagos, Nigeria. If follow-up studies confirm the high contaminant concentrations found in the present study, an immediate solution would be to change or replace deteriorating galvanised pipes that convey water to the affected residential areas.

Although, the present study sampled piped drinking water in three residential areas of Lagos, Nigeria, it should be noted that galvanised piping is widely used throughout the world. Therefore, the problem of drinking water contamination brought about by poorly maintained galvanised pipe networks could be a serious issue that transcends the boundary of Lagos, Nigeria.

## Conclusions

The high concentrations of phthalates, Cd, Pb, and Zn found in the water sampled in the present study require immediate follow-up actions and interventions by the Lagos State Government of Nigeria and the state's water corporation as high levels of these contaminants in drinking water pose real and immediate neurodevelopment risks to children and women of child-bearing age. Cadmium and Pb are cumulative poisons that induce renal dysfunction among other effects, and Zn poisoning can result in adverse health conditions including pulmonary distress, fever, chills, and gastroenteritis. Children are especially vulnerable to these adverse effects because their body's contaminants detoxifying systems are not yet fully developed.
